# Factors affecting adherence with treatment advice in a clinical trial of patients with severe asthma

**DOI:** 10.1183/13993003.00768-2021

**Published:** 2022-04-28

**Authors:** John Busby, John G. Matthews, Rekha Chaudhuri, Ian D. Pavord, Timothy C. Hardman, Joseph R. Arron, Peter Bradding, Christopher E. Brightling, David F. Choy, Douglas C. Cowan, Ratko Djukanovic, Catherine E. Hanratty, Tim W. Harrison, Cecile T. Holweg, Peter H. Howarth, Stephen J. Fowler, James L. Lordan, Adel H. Mansur, Andrew Menzies-Gow, Robert M. Niven, Douglas S. Robinson, Samantha M. Walker, Ashley Woodcock, Liam G. Heaney

**Affiliations:** 1 Centre for Experimental Medicine, School of Medicine, Dentistry and Biomedical Sciences, Queen's University Belfast, Belfast, UK; 2 23 and Me, Sunnyvale, CA, USA; 3 NHS Greater Glasgow and Clyde Health Board, Gartnavel General Hospital and University of Glasgow, Glasgow, UK; 4 Oxford Respiratory NIHR BRC, Nuffield Dept of Medicine, The University of Oxford, Oxford, UK; 5 Niche Science & Technology Ltd, London, UK; 6 Genentech Inc., South San Francisco, CA, USA; 7 Dept of Respiratory Sciences, Institute for Lung Health and Leicester NIHR Biomedical Research Centre, University of Leicester, Leicester, UK; 8 NHS Greater Glasgow and Clyde, Stobhill Hospital, Glasgow, UK; 9 School of Clinical and Experimental Sciences, University of Southampton, NIHR Southampton Biomedical Research Centre, Southampton, UK; 10 Nottingham Respiratory NIHR Biomedical Research Centre, University of Nottingham, Nottingham, UK; 11 Division of Infection, Immunity and Respiratory Medicine, School of Biological Sciences, The University of Manchester, and Manchester Academic Health Science Centre and NIHR Manchester Biomedical Research Centre, Manchester University Hospitals NHS Foundation Trust, Manchester, UK; 12 The Newcastle upon Tyne NHS Foundation Trust, Newcastle upon Tyne, UK; 13 University of Birmingham and Heartlands Hospital, University Hospitals Birmingham NHS Foundation Trust, Birmingham, UK; 14 Royal Brompton and Harefield NHS Foundation Trust, London, UK; 15 University College Hospitals NHS Foundation Trust, London, UK; 16 Asthma UK and British Lung Foundation Partnership, London, UK

## Abstract

**Background:**

Understanding why patients with severe asthma do not follow healthcare provider (HCP) advice to adjust treatment is critical to achieving personalised disease management.

**Methods:**

We reviewed patient choice to follow HCP advice to adjust asthma treatment in a UK-based randomised, controlled, single-blind (study participant), multicentre, parallel group 48-week clinical study comparing biomarker-directed treatment adjustment with standard care in severe asthma.

**Results:**

Of 1572 treatment advisories (291 participants), instructions were followed in 1377 cases (87.6%). Patients were more likely to follow advice to remain on treatment (96.7%) than to either reduce (70.3%) or increase (67.1%) their treatment, with 64% of patients following all treatment advice. Multivariate analysis associated belonging to an ethnic minority group (OR 3.10, 95% CI 1.68–5.73) and prior study medication changes (two or more changes: OR 2.77, 95% CI 1.51–5.10) with failure to follow treatment advice. In contrast, emergency room attendance in the prior year (OR 0.54, 95% CI 0.32–0.92) was associated with following treatment advice. The largest effect was seen with transition onto or off oral corticosteroids (OR 29.28, 95% CI 16.07–53.36) when compared with those requested to maintain treatment. Centre was also an important determinant regarding the likelihood of patients to follow treatment advice.

**Conclusions:**

Belonging to an ethnic minority group and multiple prior treatment adjustments were associated with not following HCP treatment advice. Patients also responded differently to HCP advice across UK specialist centres. These findings have implications for the generalisability of models of care in severe asthma and require further focused studies.

## Abstract 2

**Belonging to a minority ethnic group, multiple prior medication changes, being treated at a specific clinical centre, introduction of systemic corticosteroids and increased asthma symptoms were associated with resistance to asthma treatment modification** https://bit.ly/3gYb66S

## Introduction

Asthma treatment guidelines advocate that treatment is increased to reduce symptoms and risk of asthma exacerbations, with consideration of treatment reduction when asthma is controlled for a period of at least 3 months [1]. This strategy requires partnership between patients and healthcare professionals (HCPs) to adjust treatment when appropriate, particularly among those patients on high-dose corticosteroid treatment [2–4].

In a recent study, we investigated two strategies for adjusting corticosteroid therapy (both inhaled corticosteroids (ICS) and oral corticosteroids (OCS)) in patients with severe asthma: type 2 inflammation (T2) biomarker adjustment *versus* adjustment using current symptoms and recent asthma exacerbation history [5]. The study was designed primarily to explore the impact of corticosteroid treatment reduction in T2 biomarker-low participants. Clear clinical benefits were seen using biomarker-based treatment adjustment in patients who followed the study treatment algorithms (pre-specified per-protocol analysis), which included a greater proportion of patients on lower dose of ICS and reduced risk of exacerbation [5]. Despite these benefits, a large proportion of patients did not follow HCP recommendations to modify treatment. A reluctance to change therapy was anticipated for treatment increase, often meaning starting OCS, but not to reduce therapy in a study where patients were advised the primary aim was to reduce corticosteroid treatment.

Asthma guidelines distil an extensive scientific literature into evidence-based treatment pathways. However, effective implementation depends on patient engagement. We explored demographic and clinical factors to identify patient barriers to following HCP advice to adjust treatment.

## Methods

### Patients and study design

We performed a *post hoc* secondary analysis of data from our UK-based randomised, controlled, single-blind (study participant), multicentre, parallel group 48-week clinical study in patients with severe asthma (Global Initiative for Asthma Steps 4 and 5 classification of asthma severity) (ClinicalTrials.gov: NCT02717689) [5, 6]. The study team included a Patient Input Platform (PIP), a panel of patients with an insight into clinical trials who provided direction regarding patient needs and how to facilitate their understanding of study aims, objectives and requirements. This group, recruited by Asthma UK, was embedded in all discussions relating to study design and implementation. The protocol was reviewed and approved by the Office for Research Ethics Northern Ireland (NI0158) and obtained local National Health Service Research and Development approval for individual sites. All patients provided written informed consent for study participation.

### Study procedures

Following randomisation, patients attended the clinic every 8 weeks for review of asthma control and treatment, and the electronic case report form software processed the study algorithms to generate a treatment advisory in both treatment arms to decrease, maintain or increase treatment. In brief, we compared a composite biomarker-based adjustment of corticosteroid therapy (using a composite index of blood eosinophil count, serum periostin concentration and fractional exhaled nitric oxide (*F*_ENO_)) with adjustments in the control arm based on asthma symptoms, lung function and recent exacerbation history. The term “advisory” was specifically chosen as it was anticipated that some patients would not follow treatment advice, *e.g.* progression to OCS. In keeping with the pragmatic nature of the study, patients were permitted to stay in the study if treatment advice was not followed, although the reasons for this were recorded at the subsequent study visit. Treatment advisories which were not followed due to reasons allowed for in the study protocol (patient on lowest allowed ICS dose or low cortisol preventing prednisolone reduction) or where other external factors influenced the patient's choice to adjust treatment (clinician decision to override treatment adjustment or site logistical error) were interpreted as the patient following HCP advice (described in supplementary table E1a and b). When a treatment advisory could not be generated (primarily due to a missing biomarker measurement), a default “maintain treatment” advisory was generated.

### Statistical analyses

Descriptive statistics are presented as mean±sd, median (interquartile range) or count (percentage) as appropriate. Comparisons between patients who followed all treatment advice during the study and those who did not follow at least one advisory were made using the t-test (normally distributed variables), Mann–Whitney U-test (nonnormally distributed variables) or Chi-squared test (categorical variables). Initial univariate logistic regression models were used to assess the association for a broad range of demographic and clinical variables that could plausibly impact the patient's decision to follow treatment advisories. A final multivariate model was selected using a modified form of backward selection. Our initial models investigated all advisories combined; however, we fitted separate models estimating the probability of following a reduce, maintain or increase advisory. To investigate potential outcome misclassification (due to intentional or unintentional patient misreporting) we compared reported medication adjustment with change in T2 biomarkers, known to be highly corticosteroid sensitive [7, 8].

Supplementary analysis compared exacerbation risk among those with a disassociated symptom/biomarker profile. A subgroup of patients with low symptoms and moderate/high biomarkers was identified, as was a separate subgroup with high symptoms and moderate/low biomarkers (supplementary material). The outcome measure was time to first exacerbation within the 8-week study period with patients considered “at risk” from the date of the study visit until the day prior to the next study visit (follow-up truncated at 56 days). Comparisons were investigated using Kaplan–Meier plots, and Cox regression models adjusted for age, gender and treatment centre were used to conduct hypothesis tests. Full details of the statistical methods are provided in the supplementary material. Analyses were conducted using Stata version 16 (StataCorp, College Station, TX, USA).

## Results

Patients (n=301) undertook 1629 visits during the course of the study; of these, 25 visits had missing data. There was no information on whether or not the treatment advice was followed for 26 visits (26 patients). In six further cases it was unclear why treatment advice was not followed. Of the remaining 1572 treatment advisories issued (291 patients), 1377 (87.6%) were followed. Patients were more likely to follow advice to remain on current treatment (96.7%) than advice to either reduce (70.3%) or increase (67.1%) treatment (supplementary table E1a and b). Where treatment advisories were reported as either followed or not followed, change in individual T2 biomarkers was consistent with accurate self-reporting of treatment (supplementary table E1c).

Baseline demographic and clinical factors in patients who followed all treatment advice (n=186 (64%)) and those who decided not to follow at least one treatment advisory (n=105 (36%)) are summarised in table 1. Minority ethnic group (13.3% *versus* 4.3%; p=0.005) and higher intensity ICS dose (2418±873 *versus* 2151±608 μg beclomethasone dipropionate equivalent; p=0.002) were associated with not following treatment advice, whereas patients on maintenance OCS at study entry (41.4% *versus* 28.6%; p=0.029) and having an emergency room attendance in the 12 months before randomisation (25.8% *versus* 15.2%; p=0.037) were more likely to follow advice. There was wide variation in the way treatment advisories were followed by patients at different clinical centres (supplementary table E2 and supplementary figure E1), which may be partly related to cross-site differences in the characteristics of patients enrolled (*e.g.* ethnicity and corticosteroid treatment intensity), although differences were also seen between sites in gender, primary care asthma attendance and asthma control (supplementary table E2).

**TABLE 1:** Baseline demographic and clinical factors in the randomised population who followed all treatment advice and those who did not follow at least one treatment advisory

	**Followed treatment advice (n=186)**	**Chose not to follow treatment advice (n=105)**	**p-value**
**Age (years)**	55.6±13.7	56.1±12.4	0.7790
**Male**	58 (31.2)	41 (39.0)	0.1739
**Ethnic minority group**	8 (4.3)	14 (13.3)	0.0051
**BMI (kg·m^−2^)**	32.1±7.3	31.3±7.0	0.3568
**Smoking status**			0.5193
Never-smoker	141 (75.8)	76 (72.4)	
Ex-smoker	45 (24.2)	29 (27.6)	
**Medical history**			
Atopic disease	130 (70.3)	71 (67.6)	0.6380
Hospital admission for asthma (prior year)	34 (18.3)	21 (20.0)	0.7189
ER attendance for asthma (prior year)	48 (25.8)	16 (15.2)	0.0366
GP attendance for asthma (prior year)	107 (57.5)	50 (47.6)	0.1034
Rescue OCS (prior year)	2.0 (1.0–4.0)	2.0 (1.0–4.0)	0.8711
ER asthma admission (ever)	34 (18.3)	28 (26.7)	0.0933
Ventilated (ever)	15 (44.1)	15 (53.6)	0.4585
**Comorbidities**			
Rhinitis	128 (68.8)	73 (69.5)	0.9003
Eczema	67 (36.0)	31 (29.5)	0.2600
Nasal polyps	43 (23.1)	27 (25.7)	0.6188
Previous nasal surgery	40 (21.5)	27 (25.7)	0.4128
Oesophageal reflux	115 (61.8)	60 (57.1)	0.4331
Aspirin sensitivity	29 (15.6)	16 (15.2)	0.9362
Depression/anxiety	60 (32.3)	31 (29.5)	0.6290
Hypertension	59 (31.7)	33 (31.4)	0.9590
Osteoporosis/osteopenia	47 (25.3)	18 (17.1)	0.1100
Osteoarthritis	54 (29.0)	23 (21.9)	0.1856
Hypercholesterolaemia	39 (21.0)	14 (13.3)	0.1051
Diabetes	21 (11.3)	12 (11.4)	0.9715
Cataracts	20 (10.8)	13 (12.4)	0.6740
Obstructive sleep apnoea	11 (5.9)	6 (5.7)	0.9444
Ischaemic heart disease	9 (4.8)	3 (2.9)	0.4143
Peptic ulcer	4 (2.2)	3 (2.9)	0.7056
Stroke	2 (1.1)	4 (3.8)	0.1150
Chronic kidney disease	2 (1.1)	5 (4.8)	0.0487
Glaucoma	4 (2.2)	0 (0.0)	0.1302
Myocardial infarction	2 (1.1)	1 (1.0)	0.9206
**Lung function**			
FEV_1_ (% pred)	75.1±20.2	76.4±17.7	0.5692
FVC (% pred)	90.4±17.1	92.1±16.7	0.4325
FEV_1_/FVC	0.66±0.12	0.66±0.11	0.8747
PEF (L)	366.1±120.3	386.5±138.4	0.1934
**Laboratory values**			
Sputum eosinophils (%)	1.5 (0.4–7.0)	1.0 (0.3–8.3)	0.9419
*F*_ENO_ (ppb)	20 (13–28)	21 (13–29)	0.8503
Blood eosinophils (×10^9^ L^−1^)	0.20 (0.11–0.32)	0.24 (0.10–0.37)	0.3364
Periostin (ng·mL^−1^)	52.0±13.8	54.6±20.1	0.2021
**Corticosteroid treatment**			
Maintenance OCS user	77 (41.4)	30 (28.6)	0.0293
OCS dose (mg)	0 (0–8)	0 (0–5)	0.0519
ICS dose (µg BDP equivalent)	2151±608	2418±873	0.0024
**Questionnaires**			
ACQ-7 score	2.1±1.1	1.8±1.2	0.0875
AQLQ total score	4.8±1.3	5.0±1.5	0.5433

Data are presented as mean±sd, n (%) or median (interquartile range), unless otherwise stated. BMI: body mass index; OCS: oral corticosteroid; ER: emergency room; GP: general practitioner; FEV_1_: forced expiratory volume in 1 s; FVC: forced vital capacity; PEF: peak expiratory flow; *F*_ENO_: fractional exhaled nitric oxide; ICS: inhaled corticosteroid; BDP: beclomethasone dipropionate; ACQ: Asthma Control Questionnaire; AQLQ: Asthma Quality of Life Questionnaire.

Univariate associations with all candidate variables are provided in supplementary table E3. In multivariate analysis, belonging to an ethnic minority group (OR 3.10, 95% CI 1.68–5.73) and prior medication changes during the course of the study (two or more changes: OR 2.77, 95% CI 1.51–5.10) were associated with failure to follow treatment advice, whereas an emergency room attendance in the prior year (OR 0.54, 95% CI 0.32–0.92) was associated with following treatment advice (table 2). The largest effect in adjusting treatment was seen with transition onto or off OCS (OR 29.28, 95% CI 16.07–53.36), although patients advised to amend OCS or ICS dose (OR 11.75, 95% CI 6.97–19.78) or add/remove a long-acting muscarinic antagonist (OR 9.84, 95% CI 4.20–23.02) were also less likely to follow treatment advice than those asked to maintain treatment. Thus, after adjusting for other factors in the model, predictions suggest that 42.8% (95% CI 32.6–53.1%) of patients decided not to initiate/discontinue OCS *versus* only 3.6% (95% CI 2.2–4.9%) who decided not to maintain treatment (difference 39.3%, 95% CI 28.9–49.6%). Study centre was an important determinant regarding the likelihood of patients to follow treatment advice, in particular patients from Site B (OR 7.54, 95% CI 3.46–16.41) were much less likely to follow treatment advisories than those from other centres. For example, model predictions suggest that 28.0% (95% CI 23.1–32.9%) of advisories were not followed at Site B *versus* just 3.9% (95% CI 0.5–0.7%) at Site C (difference 24.1%, 95% CI 17.9–30.3%) after adjusting for other factors in the model.

**TABLE 2:** Multivariate analysis of factors associated with not following treatment advice (all advisories combined)

	**Univariate**		**Multivariate**	
	**OR (95% CI)**	**p-value**	**OR (95% CI)**	**p-value**
**Centre**				
Site A	Reference		Reference	
Site B	7.44 (3.43–16.14)	0.001	7.54 (3.46–16.41)	0.001
Site C	0.46 (0.15–1.44)	0.184	0.37 (0.12–1.18)	0.094
Site D	1.31 (0.46–3.74)	0.609	1.80 (0.62–5.29)	0.282
Site E	1.94 (0.84–4.49)	0.119	1.57 (0.64–3.86)	0.322
Site F	1.57 (0.62–3.97)	0.341	1.01 (0.38–2.69)	0.987
Other	2.15 (0.98–4.69)	0.056	1.39 (0.62–3.10)	0.428
**Ethnic minority group**	2.06 (0.95–4.46)	0.068	3.10 (1.68–5.73)	0.001
**Ex-smoker**	1.11 (0.69–1.80)	0.664	1.31 (0.81–2.11)	0.265
**ER visit (prior year)**	0.58 (0.32–1.04)	0.070	0.54 (0.32–0.92)	0.024
**ACQ-7 >1.5**	0.94 (0.63–1.39)	0.757	1.16 (0.79–1.70)	0.459
**Previous changes**				
0	Reference		Reference	
1	1.67 (1.08–2.59)	0.021	1.89 (1.18–3.02)	0.008
2+	2.07 (1.19–3.62)	0.010	2.77 (1.51–5.10)	0.001
**Treatment adjustment**				
Maintain treatment	Reference		Reference	
Amend ICS/OCS dose	11.58 (7.05–19.02)	0.001	11.75 (6.97–19.78)	0.001
Add/remove LAMA	9.95 (4.82–20.53)	0.001	9.84 (4.20–23.02)	0.001
Add/remove OCS	24.36 (13.62–43.58)	0.001	29.28 (16.07–53.36)	0.001

ER: emergency room; ACQ: Asthma Control Questionnaire; OCS: oral corticosteroid; ICS: inhaled corticosteroid; LAMA: long-acting muscarinic antagonist.

**FIGURE 1:**
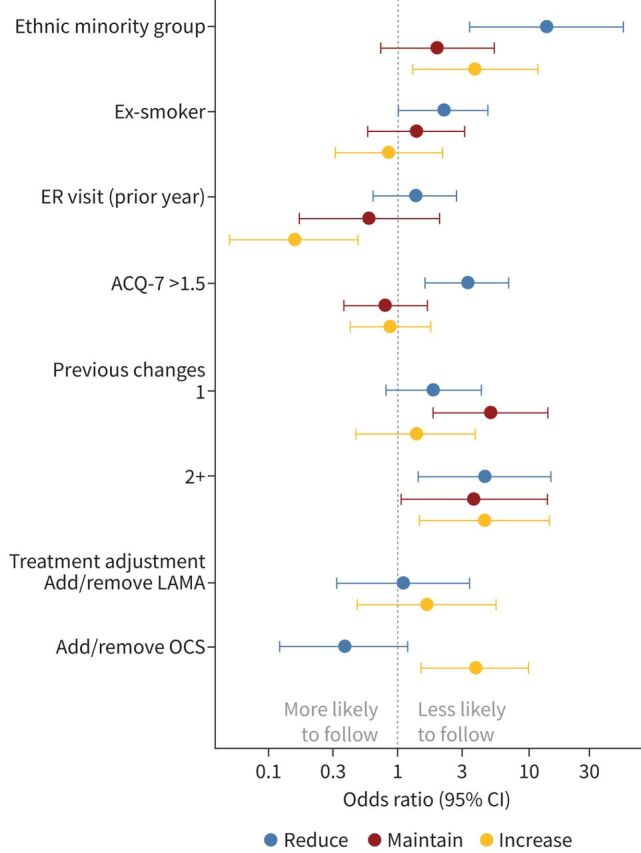
Comparison of multivariate analysis for reduce, maintain and increase advisories. ER: emergency room; ACQ: Asthma Control Questionnaire; OCS: oral corticosteroid; ICS: inhaled corticosteroid; LAMA: long-acting muscarinic antagonist.

We explored factors associated with not following treatment advice (figure 1 and supplementary table E4). Minority ethnic group and multiple prior treatment changes consistently reduced the probability of a patient following treatment adjustment across all advisories. Patients with poorer asthma control (OR 3.40, 95% CI 1.62–7.16) and ex-smokers (OR 2.23, 95% CI 1.01–4.91) were more likely to not follow reduce advisories, although there was little effect of these factors on following maintain or increase advisories. Patients were more likely to refuse initiation of OCS (OR 3.93, 95% CI 1.52–10.17), which was an anticipated predefined secondary outcome, when compared with advice to increase corticosteroid dose. Conversely, there was evidence of patients being more likely to follow treatment advice when asked to discontinue OCS (OR 0.39, 95% CI 0.13–1.19; p=0.097). Centre effects were broadly consistent across advisories to reduce, maintain or increase treatment, with patients treated at Site B least likely to follow advisories.

The diagnostic accuracy of the multivariate model for following treatment advice demonstrated an area under the curve (AUC) of 0.870 (95% CI 0.842–0.899) (supplementary figure E2), and was consistent for advice to both reduce (AUC 0.826, 95% CI 0.79–0.883) and increase (AUC 0.830, 95% CI 0.780–0.880) treatment (supplementary figure E2). The internally cross-validated AUC was similar for all analyses (*e.g.* all treatment advisories combined: AUC 0.858, 95% CI 0.814–0.882), suggesting low test error.

**FIGURE 2:**
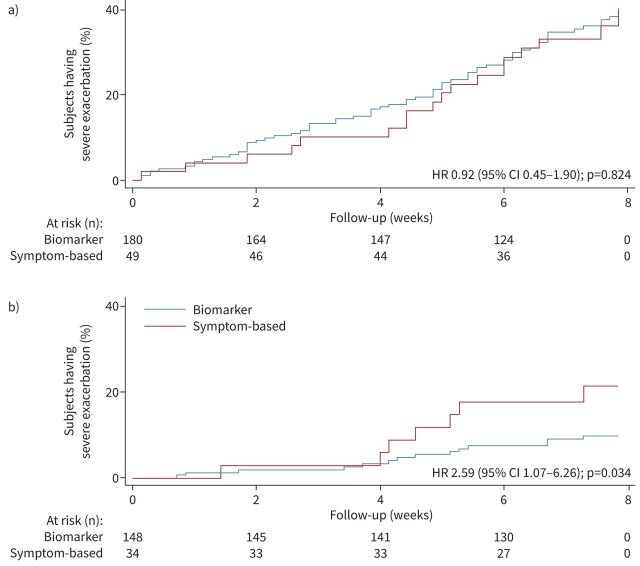
Exacerbation outcome in patients with dissociated symptoms and type 2 inflammation biomarkers. a) High symptoms/low type 2 inflammation biomarkers. We observed no benefit with increased corticosteroid dose. b) Low symptoms/high type 2 inflammation biomarkers. We observed substantial benefit with increased corticosteroid dose. HR: hazard ratio.

As current asthma symptoms impacted decisions to reduce corticosteroid treatment adversely, we analysed the impact of this decision by exploring exacerbation risk in patients where T2 biomarkers were dissociated from asthma symptoms. The symptom-based and biomarker-based algorithms are shown in supplementary table E5. Univariate analysis and demographic comparisons are shown in supplementary tables E6 and E7; in patients with high symptoms and low/moderate T2 biomarkers, exacerbation risk was no different (hazard ratio (HR) 0.92, 95% CI 0.45–1.90) when patients were managed according to symptoms (advised to increase treatment) or biomarkers (advised to maintain or reduce treatment) despite 59% of patients increasing their corticosteroid dose in the symptom-based arm *versus* only 4% in the biomarker-based arm (p<0.001) (figure 2a). However, in patients with low symptoms and moderate/high biomarkers, there was a significantly increased risk of exacerbation (HR 2.59, 95% CI 1.07–6.26) (figure 2b) when patients were managed according to symptoms (advised to reduce treatment) compared with those treated according to biomarker score (advised to maintain or increase treatment).

## Discussion

We explored reasons for patients not following HCP advice in a clinical trial comparing T2 biomarker-directed corticosteroid treatment with standard care in people with severe asthma. We reclassified any scenario where an external factor (*e.g.* advice from HCP to not follow the advice) interfered with the patient decision to follow the treatment, thus ensuring a focus on patient-directed decisions. Detailed patient information about the study explicitly described how treatment adjustment would be based on biomarker treatment in 80% taking part in the study (4:1 randomisation to T2 biomarker-directed treatment). It is generally assumed that adherence to treatment in clinical trials is high [9]; however, extensive evidence exists to the contrary [10]. The possible reluctance to initiate OCS was anticipated, thus adherence with all treatment adjustments was carefully captured at sequential study visits, allowing further analysis of the factors influencing this behaviour [5], and change in T2 biomarkers was consistent with accurate patient self-reporting of treatment.

Belonging to a minority ethnic group was consistently associated with patients not following treatment advice irrespective of type. Poorer asthma outcomes and different patterns of health service use have previously been described in UK patients with asthma from ethnic minority backgrounds [11]. It is generally accepted that ethnic diversity is inadequately reflected in clinical trials and potentially limits the applicability of results to the wider population [12, 13]. All patients were approached to take part in the clinical trial after assessment by investigators to ensure they understand the study aims and could comply with the study protocol. In this context, issues such as language barriers and comprehension of the goals of the study seem unlikely to explain ethnic differences in patient adherence to treatment guidance and further work is required to explain our observation.

Prior medication changes during the course of the study were associated with patients subsequently deciding not to follow further treatment adjustment and, importantly, this was consistent whether treatment was being increased or reduced. Where treatment is frequently adjusted, it seems logical to assume that patients decide further adjustments will not be beneficial, particularly if advice contradicts prior changes. It is also recognised that in severe asthma, more complex multimodality drug regimens are associated with treatment nonadherence [14]. Adherence with advice to maintain treatment was extremely high (97%); however, we did not predict the low level of adherence with treatment advice observed during our study and hence this is a *post hoc* analysis with no pre-specified analysis plan. Consequently, our results should be interpreted as exploratory, but future studies of biomarker-directed treatment in asthma should include detailed analysis of adherence with study advice and patient adherence to this advice. The apparent reluctance to adjust established treatment has potential implications for asthma guidelines, which currently suggest treatment reduction after periods of asthma stability and treatment increases if asthma control deteriorates [1]. It should be noted that while individual components of these guidelines have been formally tested, the overall benefit of a stepwise model of care included in asthma guidelines has not been formally validated in a controlled clinical trial. A more successful strategy, particularly in cases of more severe asthma that could progress to high-dose ICS, may be to target treatment using predictive biomarkers of therapeutic response on first presentation and maintain patients on this “correct” treatment when stable [15, 16].

Marked variation in patients following treatment advice was seen across different clinical centres (particularly one centre), which occurred consistently irrespective of the type of advice given. Some of the variability may be explained by centre differences in the patient population; it also suggests that patient willingness to adjust treatment was substantially different between centres. This heterogeneity is surprising considering the specialist nature of the centres and since the intention to adjust treatment had been communicated clearly to patients as a core study aim. It may reflect a patient's belief that they were currently on “optimal” treatment and lack of confidence that the provided advice was correct. Whatever the mechanism, it is an important observation as even under the tight constraints of a clinical trial, with standardised algorithms and training of site staff relating to treatment adjustment, patient behaviour differed markedly between clinical centres. Further work is needed to clarify why there was such disparity between centres. Prior patient experience of biomarker-directed care is a potential factor, particularly when these dissociate from symptoms. There are clearly implications around implementation of any future models of asthma care that require treatment adjustment as this may be differentially acceptable to individual patients being managed at different clinical centres.

Recent emergency room attendance was associated with patients following treatment advice, potentially as this recent “watershed” event may make patients more engaged in treatment advice and more open to the benefits of changing treatment. Poor adherence with asthma treatment has consistently been shown to be associated with increased emergency room visits [17], but our findings suggest that a prior emergency room visit is also associated with a greater willingness to increase treatment, suggesting this may be a bidirectional relationship. Not following advice to initiate OCS was anticipated, reflecting patient dislike of their well-recognised side-effects [2, 18, 19]. However, lack of engagement with treatment adjustment was evident across all therapeutic changes, consistent with a general reticence to adjust any form of treatment.

The accuracy of the multivariate model examining patient adherence with treatment advice supports our understanding that most important variables affecting patient treatment decisions were captured; however, further in-depth qualitative studies exploring the roots of these associations are required to aid the design of effective interventions. Indeed, a literature review identified six key factors that contribute to intentional nonadherence among older adults (illness beliefs, perceived treatment risks, benefits and necessity of potential treatments, patient–practitioner relationship, poly-pharmacy/regimen complexity, and inter-current physical/mental illness) and all of the factors identified in our analysis can be mapped onto these areas [20]. The study team took advice on study design from a panel of expert patients (PIP) who advised that patients would be enthusiastic about both biomarker-directed treatment and achieving low doses of corticosteroid. However, UK patient/public advisory groups in health research are often unrepresentative of the wider patient population, skewing towards those who are White, middle-class and older aged. This risks overlooking some of the key barriers to study design identified in our study and, specifically, the impact of ethnicity on patient decisions, must inform PIP selection criteria for future programmes.

As the study focused on reducing corticosteroid treatment, we explored patients’ reasons for choosing not to reduce treatment, and identified being an ex-smoker and having uncontrolled asthma as key factors. The latter finding suggests that some study participants would have benefited from a more thorough explanation of the dissociation between symptoms and corticosteroid dose when T2 biomarkers are low. As prominent asthma symptoms adversely impacted on advice to reduce corticosteroid treatment, we examined exacerbation risk in patients where T2 biomarkers were dissociated from asthma symptoms. Among those with high symptoms and low/moderate biomarkers, exacerbation risk was not different when patients were asked to reduce/maintain corticosteroid treatment based on T2 biomarkers, whereas in those with high/moderate T2 biomarkers and low symptoms, exacerbation risk was higher when biomarkers were ignored when determining the treatment advisory. This increased risk of exacerbation has been described previously in sputum-guided treatment adjustment where symptom-low/sputum eosinophil-high patients had a 10-fold reduction in exacerbation risk when treatment was increased according to sputum eosinophilia [21]. Taken together, high levels of symptoms are associated with patients deciding not to reduce treatment where this is appropriate (T2 biomarker-low) and treatment adjustment based on low symptoms (and ignoring high T2 biomarkers) is associated with increased risk. However, this study demonstrates that while T2 biomarkers provide prognostic information and correct corticosteroid treatment advice, many symptomatic patients will decide not to follow appropriate advice to reduce corticosteroid treatment.

In conclusion, we identified factors associated with patients not following HCP treatment advice within a robustly conducted randomised controlled trial, which may be important in improving patient engagement with HCP-directed advice in the routine management of severe asthma. Factors such as minority ethnic group and clinical centre require further focused studies to explore the underlying reasons for their importance. Irrespective of the outcomes, these factors have implications for the generalisability of any model of care in severe asthma.

## Supplementary material

10.1183/13993003.00768-2021.Supp1 **Please note:** supplementary material is not edited by the Editorial Office, and is uploaded as it has been supplied by the author.
Supplementary appendix ERJ-00768-2021.Supplement

## Shareable PDF

10.1183/13993003.00768-2021.Shareable1 This one-page PDF can be shared freely online.
Shareable PDF ERJ-00768-2021.Shareable
