# Supplementary material for: Factors affecting adherence with treatment advice in a clinical trial of patients with severe asthma
Source: Eur Respir J. 2022 Apr 28;59(4):2100768. doi: 10.1183/13993003.00768-2021 (PMC9202483; doi:10.1183/13993003.00768-2021)
Supplement: Supplementary file 1 [file ERJ-00768-2021.Supplement.pdf]

Supplementary Appendix

Factors affecting adherence with treatment advice in a clinical trial of patients with severe asthma.

LIST OF PARTICIPATING CLINICAL CENTRES

NHS Clinical Centres with a dedicated tertiary care in difficult asthma service that recruited to the study

- Belfast Health & Social Care Trust
- Oxford University Hospitals NHS Trust
- Glenfield Hospital, University Hospitals of Leicester NHS Trust
- Wythenshawe Hospital, University Hospitals of South Manchester NHS Trust
- University Hospital Southampton NHS Foundation Trust
- Royal Brompton & Harefield NHS Foundation Hospital
- King's College Hospital NHS Foundation Trust
- Nottingham University Hospitals NHS Foundation Trust
- Sheffield Teaching Hospitals NHS Foundation Trust
- Gartnavel and Stobhill/Glasgow Royal Infirmary Hospitals, Greater Glasgow Health Board
- Heartlands Hospital, University Hospitals Birmingham NHS Foundation Trust
- Freemans Hospital, Newcastle upon Tyne NHS Foundation Trust

List of industrial partners in RASP-UK consortium"

- GlaxoSmithKline
- Hoffman la Roche / Genentech Inc
- Amgen
- Astra Zeneca / Medimmune
- Boehringer Ingelheim
- Jannsen
- Circassia
- Vitalograph

Supplementary Table E1a. Recorded reasons for patients not adjusting treatment after an advisory

Reason	Advisory		
	Reduce	Maintain	Increase
**Patient directed decisions**			
Patient choice	47 (54.0%)	20 (48.8%)	84 (76.4%)
Asthma control deteriorated	10 (11.5%)	6 (14.6%)	2 (1.8%)
Exacerbation	8 (9.2%)	5 (12.2%)	1 (0.9%)
Patient error	4 (4.6%)	4 (9.8%)	4 (3.6%)
**External factors interfering with patient directed decision**			
Clinician Decision	8 (9.2%)	5 (12.2%)	8 (7.3%)
Logistical error	10 (11.5%)	1 (2.4%)	9 (8.2%)
GP Decision	0 (0.0%)	0 (0.0%)	2 (1.8%)

Supplementary Table E1b: Treatment advisories followed in each study arm included in analysis

Treatment Arm	Followed	Refuse
**Biomarker**		
Reduce	134 (70.5%)	56 (29.5%)
Maintain	821 (96.5%)	30 (3.5%)
Increase	139 (67.1%)	68 (32.9%)
**Symptom Assessment**		
Reduce	30 (69.8%)	13 (30.2%)
Maintain	206 (97.6%)	5 (2.4%)
Increase	47 (67.1%)	23 (32.9%)
**Overall**		
Reduce	164 (70.4%)	69 (29.6%)
Maintain	1027 (96.7%)	35 (3.3%)
Increase	186 (67.1%)	91 (32.9%)

Supplementary Table E1c Median % change in type-2 biomarkers between study visits compared to patient self-reported treatment adjustments between study visits for all subjects, including where patients chose not to follow treatment advice. The changes are consistent with expected change in biomarker profile for self-reported treatment

Biomarker	No Change in Treatment	Decreased ICS	Increased ICS	Decreased OCS	Increased OCS
FeNO	0% (-21,36); n=848	14% (-11,46); n=96	-5% (-21, 0); n=11	24% (-11,75); n=47	-17% (-43, 8); n=129
Blood Eosinophils	1% (-20,34); n=823	21% (0,77); n=95	-1% (-15,11); n=12	50% (-13,150); n=42	-47% (-71,-21); n=128
Periostin	1% (-6, 8); n=822	6% (-2,12); n=93	-4% (-13, 2); n=11	8% (-3,21); n=46	-13% (-17,-3); n=127

Supplementary Figure E1. Number of treatment advisories where patients chose not to follow treatment advice by individual clinical centres. Any treatment advisory which was not followed because it was within study protocol (patient on lowest allowed ICS dose, low cortisol preventing prednisolone reduction) or where external barriers intervened in the patient decision to follow study treatment advice (clinician decision to override treatment adjustment or site logistical error) were interpreted as patient following advice to maintain treatment.

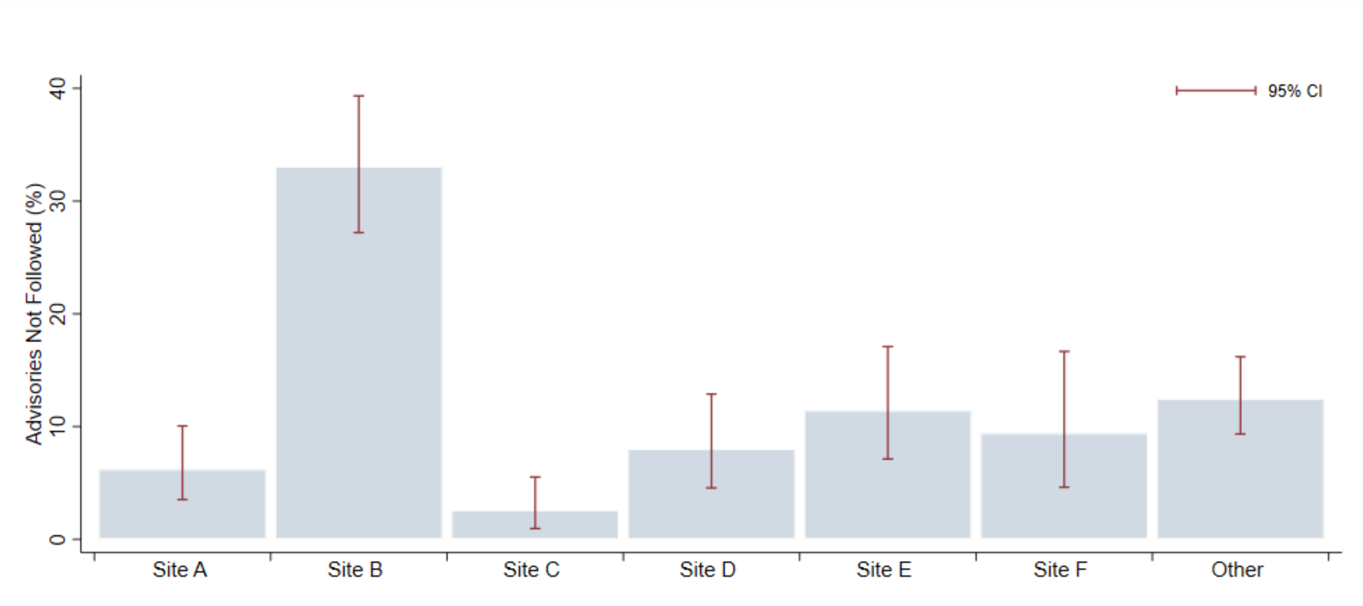

Supplementary Table E2 Demographics, medical history, comorbidities, lung function and corticosteroid treatment in the randomised population by Clinical Centre

	A	B	C	D	E	F	Others (6 centres) [n< 20 per centre]	P-value
**Number of Patients; N=291**	43	44	44	32	31	20	77	
**Advisories not followed in study**	15 (6.2%)	81 (33.1%)	6 (2.6%)	15 (8.0%)	20 (11.4%)	10 (9.4%)	48 (12.5%)	<0.0001
**Advisories not followed (patient choice)**	10 (66.7%)	71 (87.7%)	5 (83.3%)	10 (66.7%)	18 (90.0%)	5 (50.0%)	32 (66.7%)	0.0125
**Age At Inclusion; N=291**	57.7 (14.5)	56.6 (12.7)	55.9 (12.1)	57.8 (11.8)	55.5 (12.0)	54.2 (11.8)	53.8 (14.8)	0.6922
**Gender; N=291**								0.0217
Female	24 (55.8%)	21 (47.7%)	32 (72.7%)	26 (81.2%)	22 (71.0%)	16 (80.0%)	51 (66.2%)	
Male	19 (44.2%)	23 (52.3%)	12 (27.3%)	6 (18.8%)	9 (29.0%)	4 (20.0%)	26 (33.8%)	
**Ethnicity; N=291**								0.0028
White	39 (90.7%)	40 (90.9%)	43 (97.7%)	32 (100.0%)	30 (96.8%)	14 (70.0%)	71 (92.2%)	
Ethnic Minority Groups	4 (9.3%)	4 (9.1%)	1 (2.3%)	0 (0.0%)	1 (3.2%)	6 (30.0%)	6 (7.8%)	
**BMI (kg/m2); N=290**	32.7 (7.7)	30.0 (7.2)	33.5 (6.8)	30.2 (5.1)	33.2 (8.4)	32.1 (4.0)	31.4 (7.8)	0.1682
**Smoking Status; N=291**								0.9511
Never Smoked	34 (79.1%)	31 (70.5%)	34 (77.3%)	22 (68.8%)	23 (74.2%)	15 (75.0%)	58 (75.3%)	
Ex-Smoker	9 (20.9%)	13 (29.5%)	10 (22.7%)	10 (31.2%)	8 (25.8%)	5 (25.0%)	19 (24.7%)	
**Atopic Disease; N=290**	32 (74.4%)	35 (79.5%)	28 (63.6%)	18 (56.3%)	22 (71.0%)	13 (68.4%)	53 (68.8%)	0.4252
**Hospital admission for asthma (previous year); N=291**	3 (7.0%)	12 (27.3%)	6 (13.6%)	7 (21.9%)	9 (29.0%)	4 (20.0%)	14 (18.2%)	0.1681
**Emergency room attendance for asthma (previous year); N=291**	4 (9.3%)	8 (18.2%)	12 (27.3%)	7 (21.9%)	11 (35.5%)	7 (35.0%)	15 (19.5%)	0.0992
**General practice attendance for asthma (previous year); N=291**	23 (53.5%)	12 (27.3%)	28 (63.6%)	25 (78.1%)	19 (61.3%)	11 (55.0%)	39 (50.6%)	0.0009
**Rescue OCS (Last Year); N=291**	2.0 (0.0,3.0)	2.0 (1.0,3.0)	2.0 (1.0,4.0)	2.5 (1.0,4.0)	3.0 (1.0,5.0)	3.0 (1.0,4.0)	2.0 (1.0,4.0)	0.3623
**Previous ICU; N=291**	9 (20.9%)	10 (22.7%)	12 (27.3%)	6 (18.8%)	6 (19.4%)	4 (20.0%)	15 (19.5%)	0.9683
**Ever Been Ventilated; N=62**	2 (22.2%)	3 (30.0%)	9 (75.0%)	5 (83.3%)	3 (50.0%)	1 (25.0%)	7 (46.7%)	0.0866
**History Of Rhinitis; N=291**	38 (88.4%)	27 (61.4%)	28 (63.6%)	23 (71.9%)	21 (67.7%)	12 (60.0%)	52 (67.5%)	0.1114
**History Of Eczema; N=291**	11 (25.6%)	16 (36.4%)	9 (20.5%)	11 (34.4%)	7 (22.6%)	8 (40.0%)	36 (46.8%)	0.0460
**History Of Nasal Polyps; N=291**	13 (30.2%)	12 (27.3%)	13 (29.5%)	3 (9.4%)	9 (29.0%)	4 (20.0%)	16 (20.8%)	0.3494
**Previous Nasal Surgery; N=291**	11 (25.6%)	11 (25.0%)	11 (25.0%)	5 (15.6%)	5 (16.1%)	5 (25.0%)	19 (24.7%)	0.8864
**History of Oesophageal Reflux; N=291**	29 (67.4%)	24 (54.5%)	27 (61.4%)	14 (43.8%)	22 (71.0%)	12 (60.0%)	47 (61.0%)	0.3508
**History of Aspirin Sensitivity; N=291**	15 (34.9%)	4 (9.1%)	4 (9.1%)	4 (12.5%)	4 (12.9%)	4 (20.0%)	10 (13.0%)	0.0128

**Depression / Anxiety; N=291**	12 (27.9%)	12 (27.3%)	19 (43.2%)	9 (28.1%)	14 (45.2%)	8 (40.0%)	17 (22.1%)	0.1195
**Hypertension; N=291**	16 (37.2%)	12 (27.3%)	14 (31.8%)	14 (43.8%)	9 (29.0%)	5 (25.0%)	22 (28.6%)	0.6744
**Osteoporosis / Osteopenia; N=291**	9 (20.9%)	10 (22.7%)	12 (27.3%)	4 (12.5%)	13 (41.9%)	4 (20.0%)	13 (16.9%)	0.0980
**Osteoarthritis; N=291**	13 (30.2%)	9 (20.5%)	11 (25.0%)	4 (12.5%)	12 (38.7%)	6 (30.0%)	22 (28.6%)	0.3136
**Hypercholesterolaemia; N=291**	7 (16.3%)	5 (11.4%)	10 (22.7%)	3 (9.4%)	7 (22.6%)	8 (40.0%)	13 (16.9%)	0.1003
**Diabetes; N=291**	6 (14.0%)	5 (11.4%)	3 (6.8%)	2 (6.3%)	5 (16.1%)	4 (20.0%)	8 (10.4%)	0.6389
**Cataracts; N=291**	7 (16.3%)	5 (11.4%)	3 (6.8%)	2 (6.3%)	5 (16.1%)	3 (15.0%)	8 (10.4%)	0.7030
**Obstructive Sleep Apnoea; N=291**	2 (4.7%)	2 (4.5%)	4 (9.1%)	1 (3.1%)	6 (19.4%)	2 (10.0%)	0 (0.0%)	0.0085
**Ischaemic Heart Disease; N=291**	1 (2.3%)	2 (4.5%)	1 (2.3%)	0 (0.0%)	4 (12.9%)	0 (0.0%)	4 (5.2%)	0.1597
**Peptic Ulcer; N=291**	1 (2.3%)	4 (9.1%)	0 (0.0%)	0 (0.0%)	2 (6.5%)	0 (0.0%)	0 (0.0%)	0.0218
**Stroke; N=291**	1 (2.3%)	3 (6.8%)	0 (0.0%)	0 (0.0%)	2 (6.5%)	0 (0.0%)	0 (0.0%)	0.0729
**Chronic Kidney Disease; N=291**	1 (2.3%)	2 (4.5%)	1 (2.3%)	0 (0.0%)	0 (0.0%)	2 (10.0%)	1 (1.3%)	0.2585
**Glaucoma; N=291**	1 (2.3%)	0 (0.0%)	0 (0.0%)	1 (3.1%)	1 (3.2%)	1 (5.0%)	0 (0.0%)	0.4196
**Myocardial Infarction; N=291**	0 (0.0%)	1 (2.3%)	0 (0.0%)	0 (0.0%)	2 (6.5%)	0 (0.0%)	0 (0.0%)	0.0656
**% Predicted FEV1; N=291**	77.6 (19.9)	78.9 (20.2)	75.6 (19.0)	75.2 (21.9)	75.6 (18.7)	72.1 (17.7)	73.4 (18.3)	0.7507
**% Predicted FVC; N=291**	91.9 (17.3)	91.5 (16.2)	94.5 (16.4)	89.2 (15.3)	89.6 (12.7)	82.8 (16.5)	91.7 (19.4)	0.2921
**FEV_1/FVC; N=291**	0.66 (0.10)	0.67 (0.11)	0.63 (0.10)	0.66 (0.16)	0.67 (0.11)	0.70 (0.11)	0.64 (0.12)	0.2813
**PEFR (L/min); N=288**	409.9 (133.4)	406.0 (150.9)	337.4 (115.9)	346.0 (95.6)	389.0 (114.6)	335.9 (125.5)	369.7 (125.3)	0.0343
**Sputum Eosinophils (%); N=119**	1.3 (0.3,4.5)	1.7 (0.8,33.0)	1.9 (0.5,13.1)	1.0 (0.3,8.0)	5.4 (0.0,36.2)	. (.,.)	1.3 (0.3,4.8)	
**FeNo (ppb); N=291**	21 (13,31)	20 (12,28)	14 (11,24)	23 (13,29)	24 (14,38)	20 (17,28)	23 (14,29)	0.0639
**Blood Eosinophils (109/L); N=291**	0.18 (0.11,0.27)	0.23 (0.10,0.35)	0.26 (0.14,0.35)	0.18 (0.10,0.26)	0.26 (0.12,0.55)	0.23 (0.07,0.29)	0.21 (0.11,0.32)	0.1808
**Periostin (ng/ml); N=290**	52.2 (13.9)	57.1 (20.2)	54.9 (16.8)	52.0 (10.8)	51.0 (16.5)	49.5 (11.2)	52.1 (17.9)	0.5201
**OCS User; N=291**	27 (62.8%)	9 (20.5%)	26 (59.1%)	4 (12.5%)	14 (45.2%)	3 (15.0%)	24 (31.2%)	<0.0001
**OCS Dose; N=291**	5 (0,10)	0 (0,0)	5 (0,8)	0 (0,0)	0 (0,10)	0 (0,0)	0 (0,5)	0.0008
**ICS Dose (BDP); N=291**	1958 (307)	2431 (849)	2000 (0)	1938 (148)	2645 (994)	2105 (801)	2451 (855)	<0.0001
**ACQ7 Score; N=291**	2.0 (1.1)	1.5 (1.1)	2.1 (1.1)	1.9 (0.9)	2.1 (1.2)	2.7 (1.4)	1.9 (1.1)	0.0035
**AQLQ Total Score; N=281**	5.0 (1.3)	5.3 (1.5)	4.5 (1.4)	4.9 (1.2)	4.8 (1.4)	4.2 (1.7)	5.0 (1.3)	0.0546

ER = Emergency Room; ACQ = Asthma Control Questionnaire; OCS = oral corticosteroid; ICS = inhaled corticosteroid; FEV_1 = forced expiratory volume in one second; FVC = forced vital capacity; PEFR = peak expiratory flow rate

Supplementary Table E3. Univariate associations with all candidate variables used in multivariate analysis

Variable	Description	Categorisation	Univariate association with refusing advisory[a]			
			Overall	Reduce	Maintain	Increase
**Gender**	Patient gender	Female	Ref	Ref	Ref	Ref
		Male	1.05 (0.70,1.60)	0.97 (0.44,2.14)	1.13 (0.54,2.37)	1.00 (0.49,2.03)
**Age**	Age at time of study entry (years)	<50	Ref	Ref	Ref	Ref
		50-69	1.22 (0.76,1.94)	1.10 (0.55,2.18)	1.35 (0.56,3.26)	1.27 (0.60,2.68)
		70+	1.02 (0.53,1.97)	0.74 (0.24,2.27)	1.71 (0.60,4.88)	1.24 (0.43,3.59)
**Ethnicity**	Patient ethnicity	White	Ref	Ref	Ref	Ref
		Ethnic Minority Groups	2.06 (0.95,4.46)	7.85 (2.00,30.86)	1.58 (0.61,4.07)	2.85 (0.80,10.13)
**BMI**	Body mass index (kg/m^2)	<24.9	Ref	Ref	Ref	Ref
		25-29.9	1.32 (0.68,2.54)	1.81 (0.51,6.49)	0.88 (0.26,2.96)	1.97 (0.70,5.51)
		30+	0.83 (0.44,1.56)	1.25 (0.39,4.07)	0.84 (0.27,2.59)	0.79 (0.29,2.13)
**Smoking status**	Patient smoking status	Never Smoked	Ref	Ref	Ref	Ref
		Ex-Smoker	1.11 (0.69,1.80)	1.51 (0.80,2.85)	1.33 (0.59,3.00)	0.96 (0.43,2.15)
**Years since asthma diagnosis**	Time between asthma diagnosis and study entry (years)	<15	Ref	Ref	Ref	Ref
		15-29	1.04 (0.58,1.87)	1.39 (0.54,3.61)	1.22 (0.39,3.84)	0.92 (0.40,2.13)
		30+	1.10 (0.66,1.83)	1.36 (0.66,2.78)	1.18 (0.41,3.36)	1.61 (0.73,3.55)
**Asthma hospitalisation**	Hospitalisation for asthma in the previous year	No	Ref	Ref	Ref	Ref
		Yes	0.82 (0.49,1.37)	1.00 (0.48,2.08)	1.26 (0.53,2.98)	0.41 (0.13,1.28)
**ER visit**	Emergency department attendance for asthma in the previous year	No	Ref	Ref	Ref	Ref
		Yes	0.58 (0.32,1.04)	1.42 (0.60,3.36)	0.61 (0.21,1.78)	0.28 (0.09,0.85)
**ICU admission**	ICU admission for asthma ever	No	Ref	Ref	Ref	Ref
		Yes	1.75 (1.09,2.80)	2.49 (1.26,4.92)	1.08 (0.42,2.75)	1.41 (0.59,3.37)
**Comorbidities**	Number of comorbidities (oesophageal reflux, depression / anxiety, hypertension, osteoporosis / osteopenia, osteoarthritis, hypercholesterolemia, diabetes, cataracts, obstructive sleep apnoea, ischemic heart disease, stroke, peptic ulcer, chronic kidney disease, glaucoma, myocardial infarction) dichotomised as ≤3 and 4+.	≤3	Ref	Ref	Ref	Ref
		>4	0.82 (0.53,1.25)	0.95 (0.47,1.91)	1.89 (0.91,3.89)	0.71 (0.33,1.52)
**Depression / anxiety**	Diagnosis of depression / anxiety	No	Ref	Ref	Ref	Ref
		Yes	1.10 (0.68,1.78)	1.35 (0.65,2.79)	1.08 (0.51,2.30)	1.53 (0.66,3.56)
**Recent exacerbation**	Asthma exacerbation since the previous study visit	No	Ref	Ref	Ref	Ref
		Yes	1.51 (1.02,2.22)	1.80 (0.85,3.80)	1.67 (0.79,3.53)	1.12 (0.56,2.23)
**ACQ7 score**	Asthma control questionnaire at the time of the study visit	<1.5	Ref	Ref	Ref	Ref
		>1.5	0.94 (0.63,1.39)	2.65 (1.42,4.92)	0.73 (0.35,1.54)	0.63 (0.33,1.22)
**ACQ7 difference**	Change in asthma control questionnaire from the last study visit	>0.5 Improvement	Ref	Ref	Ref	Ref
		<0.5 Change	0.69 (0.45,1.05)	0.34 (0.16,0.72)	1.09 (0.41,2.86)	0.98 (0.44,2.18)

		>0.5 Deterioration	1.08 (0.69,1.69)	1.00 (0.44,2.31)	1.26 (0.37,4.26)	0.96 (0.38,2.42)
**FEV$_1$ (% Predicted)**	FEV1 (%) measurement as the time of the study visit	<60	Ref	Ref	Ref	Ref
		60-79	1.09 (0.63,1.90)	1.02 (0.34,3.03)	2.31 (0.88,6.12)	0.51 (0.21,1.27)
		80+	0.96 (0.55,1.68)	0.64 (0.24,1.73)	1.51 (0.50,4.56)	0.66 (0.28,1.54)
**Previous changes**	Number of reduce or increase advisories that have previously been followed by the patient	0	Ref	Ref	Ref	Ref
		1	1.67 (1.08,2.59)	1.60 (0.76,3.38)	6.00 (2.17,16.62)	0.72 (0.30,1.75)
		2+	2.07 (1.19,3.62)	2.62 (0.96,7.15)	3.62 (1.05,12.44)	1.41 (0.59,3.36)
**Treatment adjustment**	Change in medication that would be required if patient was to follow treatment advisory	Maintain Treatment	Ref			
		ICS/OCS Change	11.58 (7.05,19.02)	Ref		Ref
		Add/Remove LAMA	9.95 (4.82,20.53)	0.83 (0.35,1.97)		1.55 (0.54,4.45)
		Add/Remove OCS	24.36 (13.62,43.58)	0.85 (0.18,3.93)		3.23 (1.56,6.69)
		Maintain Treatment	Ref			

ER = Emergency Room; ACQ = Asthma Control Questionnaire; ICU = intensive care unit; BMI = body mass index

Supplementary Table E4. Comparison of multivariate analysis for reduce and increase advisories

	Reduce (N=233)			Maintain (N=1,062)			Increase (N=277)		
	Unadjusted OR (95% CI)	Adjusted OR (95% CI)	P-value	Unadjusted OR (95% CI)	Adjusted OR (95% CI)	P-value	Unadjusted OR (95% CI)	Adjusted OR (95% CI)	P-value
**Centre**									
Site A	Ref	Ref	.	Ref	Ref	.	Ref	Ref	.
Site B	7.65 (2.15,27.20)	11.42 (2.43,53.61)	0.002	2.68 (0.90,7.92)	3.14 (1.04,9.47)	0.042	17.85 (4.31,73.90)	13.51 (2.29,79.80)	0.004
Site C	0.18 (0.02,1.75)	0.21 (0.02,2.68)	0.231	0.36 (0.07,1.89)	0.35 (0.06,1.98)	0.237	0.66 (0.11,4.08)	0.71 (0.07,6.96)	0.771
Site D	2.97 (0.67,13.09)	4.07 (0.69,23.92)	0.121	0.21 (0.02,1.81)	0.29 (0.03,2.41)	0.251	1.45 (0.18,11.75)	1.67 (0.17,16.44)	0.658
Site E	0.62 (0.12,3.15)	0.46 (0.08,2.70)	0.392	0.79 (0.14,4.37)	0.91 (0.16,4.99)	0.910	4.06 (0.98,16.77)	4.34 (0.70,26.74)	0.114
Site F	1.42 (0.29,6.93)	1.01 (0.22,4.53)	0.992	1.22 (0.29,5.08)	1.47 (0.35,6.11)	0.596	1.78 (0.42,7.63)	0.58 (0.11,3.09)	0.527
Other	1.86 (0.51,6.78)	1.97 (0.45,8.71)	0.370	1.05 (0.31,3.50)	1.05 (0.32,3.46)	0.931	1.93 (0.47,8.02)	1.42 (0.22,9.10)	0.711
**Ethnic Minority Groups**	7.85 (2.00,30.86)	13.60 (3.53,52.39)	0.000	1.58 (0.61,4.07)	1.98 (0.73,5.37)	0.178	2.85 (0.80,10.13)	3.88 (1.28,11.72)	0.016
**Ex-Smoker**	1.51 (0.80,2.85)	2.23 (1.01,4.91)	0.047	1.33 (0.59,3.00)	1.36 (0.57,3.20)	0.487	0.96 (0.43,2.15)	0.85 (0.33,2.20)	0.733
**ER Visit (Last Year)**	2.49 (1.26,4.92)	1.64 (0.67,4.06)	0.282	1.08 (0.42,2.74)	1.13 (0.39,3.21)	0.825	1.41 (0.59,3.37)	1.91 (0.78,4.65)	0.155
**ACQ7>1.5**	2.65 (1.42,4.92)	3.40 (1.62,7.16)	0.001	0.73 (0.35,1.54)	0.80 (0.38,1.67)	0.554	0.63 (0.33,1.22)	0.88 (0.43,1.79)	0.722
**Previous Changes**									
0	Ref	Ref	.	Ref	Ref	.	Ref	Ref	.
1	1.60 (0.76,3.38)	1.86 (0.80,4.33)	0.151	6.00 (2.17,16.62)	5.12 (1.86,14.14)	0.002	0.72 (0.30,1.75)	1.35 (0.47,3.87)	0.572
2+	2.62 (0.96,7.15)	4.63 (1.43,14.95)	0.011	3.62 (1.05,12.44)	3.84 (1.06,13.95)	0.041	1.41 (0.59,3.36)	4.61 (1.47,14.47)	0.009
**Treatment Adjustment**									
Change ICS/OCS Dose	Ref	Ref	.			.	Ref	Ref	.
Change LAMA	0.83 (0.35,1.97)	1.09 (0.34,3.53)	0.887			.	1.55 (0.54,4.45)	1.67 (0.49,5.63)	0.411
Change OCS	0.85 (0.18,3.93)	0.39 (0.13,1.19)	0.097			.	3.23 (1.56,6.69)	3.93 (1.52,10.17)	0.005

ER = Emergency Room; ACQ = Asthma Control Questionnaire; OCS = oral corticosteroid; ICS = inhaled corticosteroid; LAMA = long-acting muscarinic antagonist

Supplementary Figure E2: ROC curve for predicting refusal in all advisories combined

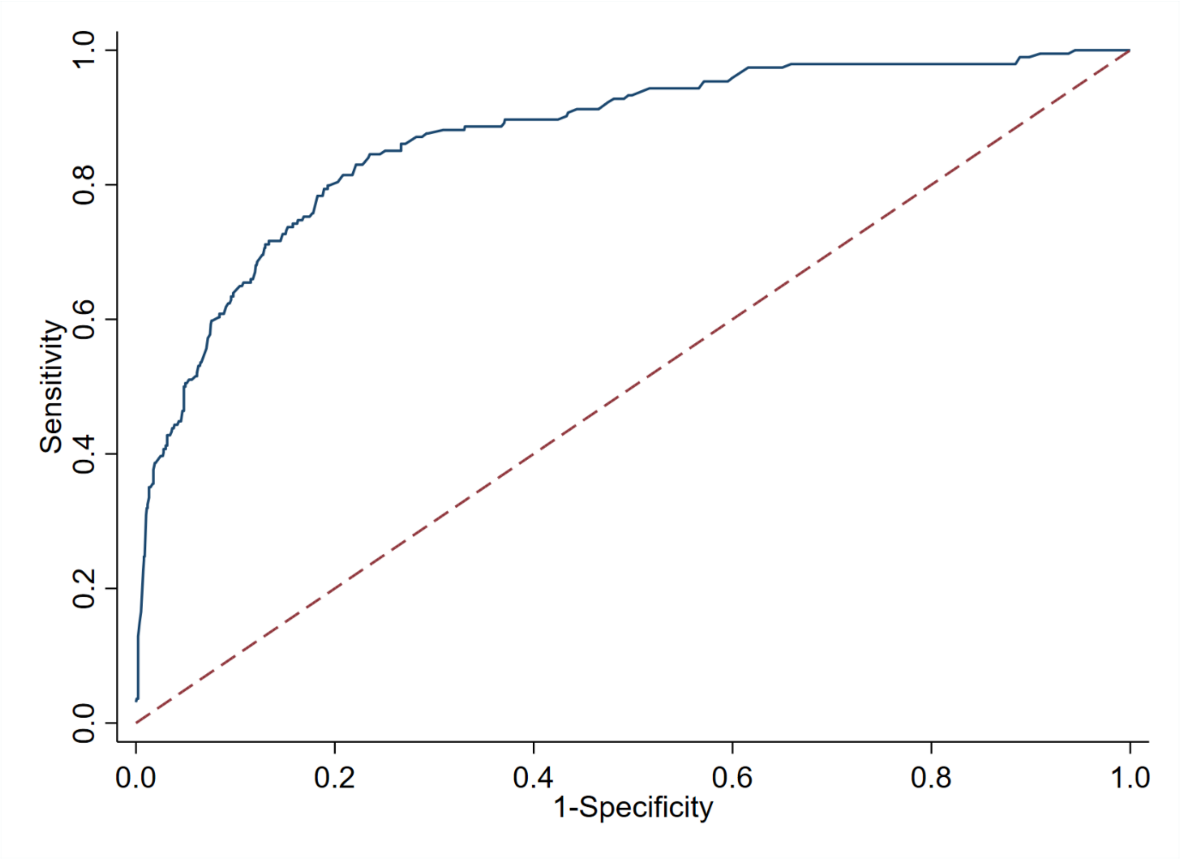

Area under the curve = 0.870 [95%: 0.842, 0899]

Supplementary Table E5. Scoring system and treatment adjustment in both study arms – treatment algorithms were generated automatically by the eCRF software – *biomarker treatment adjustment* (table E5a) – FeNO, blood eosinophil count and serum periostin were measured at each study visit with each biomarker assigned a score of 0, 1 or 2 – the composite biomarker score was generated using the rounded average of the sum of all three biomarker scores. A composite biomarker score of 0 advised treatment reduction, a score of 1 advised maintenance of current treatment and a score of 2 advised treatment increase; *symptom-/risk-based adjustment* (table E5b) – was made using the below algorithm and all therapeutic adjustments calculated automatically and advised through the e-CRF. A score of 0 advised treatment reduction, a score of 1 advised maintenance of current treatment and a score of 2 advised treatment increase. To mirror usual clinical care, patients were not asked to withhold bronchodilator medication prior to study spirometry measurements.

Supplementary Table E5a

**Scoring system**	**0**	**1**	**2**
**FeNO (ppb)**	<15	15 - 30	>30
**Blood eosinophil count (N/μL)**	< 150	150-300	>300
**Periostin (ng/mL)**	<45	45-55	>55
The **composite biomarker score** was generated using the rounded average of the sum of all three individual biomarker scores e.g. 0 + 1 + 1 = 2/3 = rounded score = 1			

FeNO = fractional exhaled nitric oxide

Supplementary Table E5b

**Asthma Control (ACQ- 7)**	**Score**
ACQ-7 ≥1.5 and ≥1 change from baseline score *OR* a severe exacerbation since last visit (past 8 weeks at baseline randomisation visit)	2
ACQ-7 is 1.0 to <1.5 OR ACQ ≥1.5 and <1 change from baseline score *AND no* severe exacerbation since last study visit (past 8 weeks at baseline randomisation visit)	1
ACQ-7 <1.0 *AND* no severe exacerbation since last study visit (prior 8 weeks at baseline randomisation visit)	0

ACQ = Asthma Control Questionnaire

Supplementary Table E6: Comparison of biomarker and symptom based algorithm at each study visit

		Biomarker[b]		
		**Score 0**	**Score 1**	**Score 2**
**Symptoms[a]**	**Score 0**	44	169	69
	**Score 1**	161	596	149
	**Score 2**	84	224	56

Low Symptoms with dissociated biomarkers (Symptoms Score 0 / Biomarker Score 1 and Score 2 cells)

High Symptoms with dissociated biomarkers (Symptoms Score 2 / Biomarker Score 0 and Score 1 cells)

[a] Symptoms Score 0 : ACQ-7 <1·0 and no severe exacerbation since last study visit (previous 8 weeks at baseline randomisation visit); Score 1: ACQ-7 is 1·0 to <1·5 or ACQ-7 ≥1·5 and <1 change from baseline score AND no severe exacerbation since last study visit (previous 8 weeks at baseline randomisation visit); Score 2: ACQ-7 ≥1·5 and ≥1 change from baseline score or a severe exacerbation since last study visit (previous 8 weeks at baseline randomisation visit)

[b] FENO, blood eosinophil count, and serum periostin were measured at each study visit with each biomarker assigned a score of 0, 1, or 2. The composite biomarker score was calculated using the rounded average of the sum of all three biomarker scores. FeNO score 0: <15 ppb, score 1: 15-30 ppb, score 2: >30 ppb. Blood eosinophil count score 0: <150 n/µL, score 1: 150-300, score 2: >300. Periostin score 0: <45ng/mL; score 1: 45-55ng/mL; score 2: >55ng/mL.

Supplementary Table E7: Demographics, medical history, comorbidities, lung function and corticosteroid treatment of patients at their first study visit with dissociated symptoms and biomarkers

	High Symptoms / Low or Moderate Biomarkers	Low Symptoms / High or Moderate Biomarkers	P-value
**Number of Patients; N=216**	133	83	
**Age At Inclusion; N=216**	53.6 (13.2)	58.0 (12.9)	0.0186
**Gender; N=216**			0.0056
Female	98 (73.7%)	46 (55.4%)	
Male	35 (26.3%)	37 (44.6%)	
**Ethnicity; N=216**			0.5397
White	122 (91.7%)	78 (94.0%)	
Ethnic Minority Groups	11 (8.3%)	5 (6.0%)	
**BMI (kg/m2); N=59**	34.3 (9.7)	28.2 (6.3)	0.0066
**Smoking Status; N=216**			0.3233
Never Smoked	104 (78.2%)	60 (72.3%)	
Ex-Smoker	29 (21.8%)	23 (27.7%)	
**Atopic Disease; N=215**	91 (68.9%)	58 (69.9%)	0.8843
**Hospital Admissions For Asthma In Last Year (Any); N=216**	27 (20.3%)	15 (18.1%)	0.6873
**A&E Visits In Last Year (Any); N=216**	32 (24.1%)	14 (16.9%)	0.2091
**GP Visits For Asthma In The Last Year (Any); N=216**	89 (66.9%)	26 (31.3%)	<0.0001
**Rescue Courses Of Oral Steroids In The Last Year; N=216**	3.0 (1.0,4.0)	2.0 (0.0,3.0)	0.0002
**Prior ICU; N=216**	33 (24.8%)	11 (13.3%)	0.0402
**Ever Been Ventilated; N=44**	18 (54.5%)	4 (36.4%)	0.2963
**History Of Rhinitis; N=216**	87 (65.4%)	59 (71.1%)	0.3864
**History Of Eczema; N=216**	47 (35.3%)	24 (28.9%)	0.3283
**History Of Nasal Polyps; N=216**	28 (21.1%)	22 (26.5%)	0.3553
**Prior Nasal Surgery; N=216**	21 (15.8%)	24 (28.9%)	0.0209
**History of Oesophageal Reflux; N=216**	83 (62.4%)	45 (54.2%)	0.2335
**History of Aspirin Sensitivity; N=216**	22 (16.5%)	10 (12.0%)	0.3659
**Depression / Anxiety; N=216**	50 (37.6%)	16 (19.3%)	0.0045
**Hypertension; N=216**	45 (33.8%)	26 (31.3%)	0.7025
**Osteoporosis / Osteopenia; N=216**	33 (24.8%)	9 (10.8%)	0.0116
**Osteoarthritis; N=216**	45 (33.8%)	15 (18.1%)	0.0119
**Hypercholesterolaemia; N=216**	23 (17.3%)	15 (18.1%)	0.8837
**Diabetes; N=216**	17 (12.8%)	8 (9.6%)	0.4824
**Cataracts; N=216**	12 (9.0%)	11 (13.3%)	0.3269
**Obstructive Sleep Apnoea; N=216**	9 (6.8%)	3 (3.6%)	0.3252
**Ischaemic Heart Disease; N=216**	5 (3.8%)	4 (4.8%)	0.7046
**Peptic Ulcer; N=216**	4 (3.0%)	1 (1.2%)	0.3914
**Stroke; N=216**	4 (3.0%)	2 (2.4%)	0.7948
**Chronic Kidney Disease; N=216**	2 (1.5%)	2 (2.4%)	0.6310
**Glaucoma; N=216**	3 (2.3%)	1 (1.2%)	0.5774
**Myocardial Infarction; N=216**	2 (1.5%)	0 (0.0%)	0.2617
**% Predicted FEV1; N=214**	68.9 (18.7)	85.3 (16.4)	<0.0001
**% Predicted FVC; N=214**	83.2 (16.4)	100.8 (15.8)	<0.0001
**FEV1/FVC; N=214**	0.66 (0.13)	0.67 (0.11)	0.6729
**PEFR (L/min); N=211**	343.5 (120.0)	424.7 (116.9)	<0.0001
**Sputum Eosinophils (%); N=30**	0.2 (0.0,0.6)	4.8 (0.5,25.5)	0.0103
**FeNo (ppb); N=216**	16 (12,27)	22 (16,32)	0.0084
**Blood Eosinophils (109/L); N=216**	0.17 (0.08,0.27)	0.21 (0.16,0.33)	0.0012
**Periostin (ng/ml); N=216**	46.0 (12.4)	58.1 (16.8)	<0.0001
**OCS User; N=216**	60 (45.1%)	31 (37.3%)	0.2610
**OCS Dose; N=216**	0 (0,10)	0 (0,5)	0.1854
**ICS Dose (BDP); N=216**	2130 (879)	2086 (750)	0.7085
**ACQ7 Score; N=215**	2.8 (1.2)	0.6 (0.2)	<0.0001
**AQLQ Total Score; N=56**	4.4 (1.5)	6.5 (0.4)	<0.0001

List of Investigators and Contributors

SITE	
Centre for Experimental Medicine, School of Medicine, Dentistry and Biomedical Sciences, Queen's University, Belfast, UK	Prof LG Heaney MD<br>Dr J Busby PhD<br>Dr CE Hanratty PhD
23andMe, Sunnyvale, California, USA	J Matthews MD
Oxford Respiratory NIHR BRC, Nuffield Department of Medicine, The University of Oxford, Oxford, UK	Prof ID Pavord FMedSci<br>Ms C Borg, BA<br>Ms C Connolly, BNS
Genentech Inc., South San Francisco, California, USA	Dr JR Arron MD<br>Dr CTJ Holweg PhD<br>Mr DF Choy BS
Department of Respiratory Sciences, Institute for Lung Health and Leicester NIHR Biomedical Research Centre, University of Leicester, Leicester, UK	Prof P Bradding DM<br>Prof CE Brightling PhD<br>Ms P McCourt RN<br>Ms B Hargadon BSc
NHS Greater Glasgow and Clyde Health Board, Gartnavel Hospital and University of Glasgow (RC), Glasgow, UK	Prof R Chaudhuri MD<br>F Yang MRCP<br>Ms T Grandison BSc
NHS Greater Glasgow and Clyde, Stobhill Hospital, Glasgow, UK	Dr DC Cowan MD<br>Ms F Steffensen BSc
Clinical and Experimental Sciences, University of Southampton and NIHR Southampton Biomedical Research Centre, Southampton, UK	Prof PH Howarth MD
Division of Infection, Immunity and Respiratory Medicine, School of Biological Sciences, The University of Manchester; Manchester Academic Health Science Centre and NIHR Manchester Biomedical Research Centre, Manchester University Hospitals NHS Foundation Trust, Manchester, UK	Prof A Woodcock MD<br>Dr SJ Fowler MD<br>Dr R Niven MD<br>Ms S Leech
Niche Science & Technology Unit 26, Falstaff House, Bardolph Road, Richmond TW9 2LH, UK	Dr TC Hardman PhD<br>Dr A Horn PhD<br>Mrs G Gainsborough BSc
Nottingham Respiratory NIHR Biomedical Research Centre, University of Nottingham, Nottingham, UK	Prof T Harrison MD<br>Prof D Shaw MD<br>Ms K Smith BSc<br>Dr H Lee PhD
Clinical and Experimental Sciences, University of Southampton, NIHR	Prof R Djukanovic MD<br>Prof P Howarth MD

Southampton Biomedical Research Centre, Southampton, UK	Dr A Azim MD
The Newcastle upon Tyne NHS Foundation Trust, Newcastle upon Tyne, UK	Dr J Lordan MD<br>Mr G Davies BSc
University of Birmingham and Heartlands Hospital, University Hospitals Birmingham NHS Foundation Trust, Birmingham, UK	Dr AH Mansur PhD<br>M Bellamy RSN<br>Dr S Davies MD
Royal Brompton & Harefield NHS Foundation Trust, London, UK	Prof A Menzies-Gow MD<br>Ms D John<br>Ms M Nunez
University College Hospitals NHS Foundation Trust, London, UK	Prof D Robinson MD<br>Mr J Solis
Asthma UK & British Lung Foundation partnership, 18 Mansell Street, London, UK	Dr SM Walker PhD
Guy's Severe Asthma Centre, Guy's Hospital, Guy's & St. Thomas' NHS Foundation Trust, London, UK	Dr C Corrigan MD<br>Dr D Jackson MD

Statistical analyses

Descriptive statistics are presented as means (SD), medians [IQR] or counts (%) as appropriate. Comparisons between patients who followed all treatment advice during the study and those who refused at least one advisory were made using the t-test (normally-distributed variables), Mann-Whitney U test (non-normally distributed variables) and chi-square test (categorical variables). Initial univariate logistic regression models were used to assess the association for a broad range of demographic and clinical variables which could plausibly impact the patient's decision to follow treatment advisories. A final multivariate model was selected using a modified form of backward selection. Our initial models investigated all advisories combined, however we fitted separate models estimating the probability of following a reduce, maintain or increase advisory. To investigate potential outcome misclassification (due to intentional or unintentional patient misreport) we compared reported medication adjustment with change in T2-biomarkers, which are known to be highly corticosteroid sensitive.

Our initial model investigated all advisories combined, however we fitted separate models estimating the probability of following a reduce, maintain or increase advisory. For simplicity, we aimed to have a consistent set of models across all analyses and so factors that were strongly prognostic for a specific advisory (e.g., increase treatment) were included in all models even if their association was weaker in other analyses. Centre effects were accounted for using fixed-effects and cluster robust standard errors were used to account for the same patients receiving multiple advisories. To improve the interpretability of our results we calculated the estimated marginal means (with 95% confidence intervals) of selected variables, adjusted for potential confounders.

Model discrimination was assessed using receiver operating characteristic (ROC) curves, and the discriminatory performance was quantified using the area under the curve (AUC). We assessed bias

using 10-fold internal cross validation. To investigate potential outcome misclassification (due to intentional or unintentional patient misreport) we compared reported medication adjustment with change in T2-biomarkers, which are known to be highly corticosteroid sensitive. For each treatment advisory we calculated the percentage change in blood eosinophils, FeNO and periostin at the subsequent visit and presented medians (IQR) separately for patients who reported decreasing ICS, decreasing oral corticosteroids (OCS), increasing ICS or increasing OCS. Visits which were preceded by an exacerbation within 14 days were excluded to negate the transient impact of rescue steroids on T2 biomarker levels.

Supplementary analysis compared exacerbation risk among those with a disassociated symptom/biomarker profile. A subgroup of patients with low symptoms and moderate/high biomarkers was identified as was a separate subgroup with high symptoms and moderate/low biomarkers (see supplementary appendix). The outcome was the time to the first exacerbation within the 8-week study period (defined as at least a doubling of treatment with OCS days [for subjects on maintenance OCS] or increase in treatment with OCS to the usual rescue course of oral steroids for ≥3 consecutive days, asthma hospitalisation or parenteral steroid use) with patients considered 'at risk' from the date of the study visit until the day prior to their next study visit (follow-up truncated at 56 days). Comparisons are displayed graphically using Kaplan-Meir plots, and Cox regression models adjusted for age, gender and treatment centre were used to conduct hypothesis tests. Cluster-robust standard errors were used to account for the same patients being included in the analysis multiple times. Analyses were conducted using STATA 16 (StataCorp, Texas, USA).

For simplicity, we aimed to have a consistent set of models across all analyses and so factors that were strongly prognostic for a specific advisory (e.g., increase treatment) were included in all models even if their association was weaker in other analyses. Centre effects were accounted for using fixed-effects and cluster robust standard errors were used to account for the same patients receiving multiple advisories. To improve the interpretability of our results we calculated the estimated marginal means (with 95% confidence intervals) of selected variables, adjusted for potential confounders [7]. Model discrimination was assessed using receiver operating characteristic (ROC) curves, and the discriminatory performance was quantified using the area under the curve (AUC). We assessed bias using 10-fold internal cross validation. To investigate potential outcome misclassification we calculated the percentage change in blood eosinophils, FeNO and periostin and presented medians (IQR) separately for patients who reported decreasing ICS, decreasing oral corticosteroids (OCS), increasing ICS or increasing OCS. Visits which were preceded by an exacerbation within 14 days were excluded to negate the transient impact of rescue steroids on T2 biomarker levels.

Supplementary analysis compared exacerbation risk among those with a disassociated symptom/biomarker profile exacerbations were defined as defined as at least a doubling of treatment with OCS days [for subjects on maintenance OCS] or increase in treatment with OCS to the usual rescue course of oral steroids for ≥3 consecutive days, asthma hospitalisation or parenteral steroid use. Cluster-robust standard errors were used to account for the same patients being included in the analysis multiple times
