# Supplementary material for: Factors affecting adherence with treatment advice in a clinical trial of patients with severe asthma
Source: Eur Respir J. 2022 Apr 28;59(4):2100768. doi: 10.1183/13993003.00768-2021 (PMC9202483; doi:10.1183/13993003.00768-2021)
Supplement: Supplementary file 2 [file ERJ-00768-2021.Shareable.pdf]

EUROPEAN RESPIRATORY JOURNAL
ORIGINAL RESEARCH ARTICLE
J. BUSBY ET AL.

SHAREABLE PDF

Factors affecting adherence with treatment advice in a clinical trial of patients with severe asthma

John Busby[1], John G. Matthews[2], Rekha Chaudhuri[3], Ian D. Pavord[4], Timothy C. Hardman[5], Joseph R. Arron[6], Peter Bradding[7], Christopher E. Brightling[7], David F. Choy[6], Douglas C. Cowan[8], Ratko Djukanovic[9], Catherine E. Hanratty[1], Tim W. Harrison[10], Cecile T. Holweg[6], Peter H. Howarth[9], Stephen J. Fowler[11], James L. Lordan[12], Adel H. Mansur[13], Andrew Menzies-Gow[14], Robert M. Niven[11], Douglas S. Robinson[15], Samantha M. Walker[16], Ashley Woodcock[11] and Liam G. Heaney[1] on behalf of the investigators for the MRC Refractory Asthma Stratification Programme

[1]Centre for Experimental Medicine, School of Medicine, Dentistry and Biomedical Sciences, Queen's University Belfast, Belfast, UK. [2]23 and Me, Sunnyvale, CA, USA. [3]NHS Greater Glasgow and Clyde Health Board, Gartnavel General Hospital and University of Glasgow, Glasgow, UK. [4]Oxford Respiratory NIHR BRC, Nuffield Dept of Medicine, The University of Oxford, Oxford, UK. [5]Niche Science & Technology Ltd, London, UK. [6]Genentech Inc., South San Francisco, CA, USA. [7]Dept of Respiratory Sciences, Institute for Lung Health and Leicester NIHR Biomedical Research Centre, University of Leicester, Leicester, UK. [8]NHS Greater Glasgow and Clyde, Stobhill Hospital, Glasgow, UK. [9]School of Clinical and Experimental Sciences, University of Southampton, NIHR Southampton Biomedical Research Centre, Southampton, UK. [10]Nottingham Respiratory NIHR Biomedical Research Centre, University of Nottingham, Nottingham, UK. [11]Division of Infection, Immunity and Respiratory Medicine, School of Biological Sciences, The University of Manchester, and Manchester Academic Health Science Centre and NIHR Manchester Biomedical Research Centre, Manchester University Hospitals NHS Foundation Trust, Manchester, UK. [12]The Newcastle upon Tyne NHS Foundation Trust, Newcastle upon Tyne, UK. [13]University of Birmingham and Heartlands Hospital, University Hospitals Birmingham NHS Foundation Trust, Birmingham, UK. [14]Royal Brompton and Harefield NHS Foundation Trust, London, UK. [15]University College Hospitals NHS Foundation Trust, London, UK. [16]Asthma UK and British Lung Foundation Partnership, London, UK.

Corresponding author: Liam G. Heaney (l.heaney@qub.ac.uk)

Check for updates

Shareable abstract (@ERSpublications)
Belonging to a minority ethnic group, multiple prior medication changes, being treated at a specific clinical centre, introduction of systemic corticosteroids and increased asthma symptoms were associated with resistance to asthma treatment modification https://bit.ly/3gYb66S

Cite this article as: Busby J, Matthews JG, Chaudhuri R, *et al*. Factors affecting adherence with treatment advice in a clinical trial of patients with severe asthma. *Eur Respir J* 2022; 59: 2100768 [DOI: 10.1183/13993003.00768-2021].

This single-page version can be shared freely online.

Copyright ©The authors 2022.

This version is distributed under the terms of the Creative Commons Attribution Licence 4.0.

Received: 15 March 2021
Accepted: 24 Aug 2021

Abstract

Background Understanding why patients with severe asthma do not follow healthcare provider (HCP) advice to adjust treatment is critical to achieving personalised disease management.

Methods We reviewed patient choice to follow HCP advice to adjust asthma treatment in a UK-based randomised, controlled, single-blind (study participant), multicentre, parallel group 48-week clinical study comparing biomarker-directed treatment adjustment with standard care in severe asthma.

Results Of 1572 treatment advisories (291 participants), instructions were followed in 1377 cases (87.6%). Patients were more likely to follow advice to remain on treatment (96.7%) than to either reduce (70.3%) or increase (67.1%) their treatment, with 64% of patients following all treatment advice. Multivariate analysis associated belonging to an ethnic minority group (OR 3.10, 95% CI 1.68–5.73) and prior study medication changes (two or more changes: OR 2.77, 95% CI 1.51–5.10) with failure to follow treatment advice. In contrast, emergency room attendance in the prior year (OR 0.54, 95% CI 0.32–0.92) was associated with following treatment advice. The largest effect was seen with transition onto or off oral corticosteroids (OR 29.28, 95% CI 16.07–53.36) when compared with those requested to maintain treatment. Centre was also an important determinant regarding the likelihood of patients to follow treatment advice.

Conclusions Belonging to an ethnic minority group and multiple prior treatment adjustments were associated with not following HCP treatment advice. Patients also responded differently to HCP advice

across UK specialist centres. These findings have implications for the generalisability of models of care in severe asthma and require further focused studies.
